# Bacterial Exposure at the Larval Stage Induced Sexual Immune Dimorphism and Priming in Adult *Aedes aegypti* Mosquitoes

**DOI:** 10.1371/journal.pone.0133240

**Published:** 2015-07-16

**Authors:** Miguel Moreno-García, Valeria Vargas, Inci Ramírez-Bello, Guadalupe Hernández-Martínez, Humberto Lanz-Mendoza

**Affiliations:** 1 Centro de Investigaciones Sobre Enfermedades Infecciosas, Instituto Nacional de Salud Pública, Av. Universidad 655. Sta. María Ahuacatitlán, 62100, Cuernavaca, Morelos, México; 2 Posgrado en Ciencias Biológicas, Universidad Nacional Autónoma de México, Edificio B, 1° Piso, Circuito de Posgrados, Ciudad Universitaria, Coyoacán 04510, México D.F; Kansas State University, UNITED STATES

## Abstract

Gender differences in the immune response of insects are driven by natural selection for females and sexual selection for males. These natural forces entail a multitude of extrinsic and intrinsic factors involved in a genotype-environment interaction that results in sex-biased expression of the genes shared by males and females. However, little is known about how an infection at a particular ontogenetic stage may influence later stages, or how it may impact sexual immune dimorphism. Using *Aedes aegypti* mosquitoes, the aim of the present study was to analyze the effect of a bacterial exposure at the larval stage on adult immunity in males and females. The parameters measured were phenoloxidase activity, nitric oxide production, antimicrobial activity, and the antimicrobial peptide transcript response. As a measure of the immune response success, the persistence of injected bacteria was also evaluated. The results show that males, as well as females, were able to enhance survival in the adult stage as a result of being exposed at the larval stage, which indicates a priming effect. Moreover, there was a differential gender immune response, evidenced by higher PO activity in males as well as higher NO production and greater antimicrobial activity in females. The greater bacterial persistence in females suggests a gender-specific strategy for protection after a previous experience with an elicitor. Hence, this study provides a primary characterization of the complex and gender-specific immune response of male and female adults against a bacterial challenge in mosquitoes primed at an early ontogenetic stage.

## Introduction

Although males and females share the genes that control general aspects of physiology, these show sex-biased expression during development. An organism develops a given phenotype because of the interaction of its genes with a multitude of factors, both extrinsic (e.g. diet, availability of food, parasites, bacteria) and intrinsic (e.g. trade-offs) [1–4]. Consequently, various ontogenetic events (e.g. food supply, infection) can have an impact on the life-history traits of adults, including fecundity and survival (reviewed in [5]).

The immune response is closely linked to survival and reproduction [6, 7], as well as being strongly influenced by these same intrinsic and extrinsic factors during development. For example, a variation in the food supply, which represents a stressful environment, can induce a less efficient immune response [8–10]. Infections during ontogeny may also have significant consequences for adult survival [11–13].

In dipterans, a previous experience with an elicitor induces an enhancement in the immune response upon re-exposure to the same agent (i.e. immune priming) [14–17]. Repeated exposure to pathogens further enhances the immune response, an effect that probably persists during the host’s lifetime and perhaps in the offspring (i.e. transgenerational priming) [18–20]. However, scant evidence exists about whether such enhanced immunity can persist from early developmental instars to adulthood (but see [13, 21, 22]), especially in holometabolous insects.

Sex differences in immunocompetence and susceptibility to pathogens have been observed in different insect groups [23, 24]. These differences are mainly attributed to the dissimilar roles of males and females in reproduction, implying that selection induces distinct patterns of investment in the immune defense. Whereas males are expected to increase their fitness through a greater investment of their resources in reproductive effort versus immune defense [25, 26], females should favor investment in immunity because increased longevity is associated with a prolonged reproductive period [27]. Contrarily, the immunocompetence of males usually decreases during an infection, supporting the hypothesis that males trade-off immune response for reproductive opportunities [23, 28]. Studies on the effect that infections at early life stages have on adult immunity may be important for understanding these gender differences.

There are also reports on the effect of the genotype-environment interaction on immunity and sexual immune dimorphism (SID) for mosquitoes. For example, a food shortage induces a weak immune response in mosquitoes [29] and infections during different ontogenetic stages may have significant consequences for adult survival [11–13]. Another study on mosquitoes found that when diets of different quality were given to larvae, dissimilar immune responses were induced in males and females [30], suggesting that each sex may use different immune strategies.

Larvae of mosquitoes, including the *Aedes spp* and *Anopheles spp* species, live in water-filled containers and feed on detritus particles, algae and bacteria [31, 32]. Whereas these bacteria may exert significant effects on the population dynamics of larvae and adult mosquitoes [33], their possible effect on the immune response of adults is unknown. Information in this respect is particularly important in respect to *Aedes aegypti*, as this species is the principal vector of the dengue fever and yellow fever viruses, which in many regions of the world are a major public health issue.

In the last few decades, knowledge of immunity-related genes and immune response pathways of mosquitoes has been rapidly expanding [34]. However, most studies have analyzed the female rather than male response to infections. Research on males could help to better understand the biology of mosquitoes and in this way facilitate genetic control in populations [35].

Hence, the aim of the present study was to analyze whether adult SID is influenced by an immune challenge during an early developmental stage. We determined whether exposure of *Ae*. *aegypti* to a bacterium at the larval stage enhanced adult immunity and/or led to immune differences between males and females (when injected with the same bacteria). Since *Escherichia coli* has been found in *Ae*. *aegypti* breeding areas in the wild [33], it was chosen for use in this study. Additionally, it has been commonly used as a pathogen model to identify immune-related genes and effectors (humoral and cellular) in adult mosquitoes [36–40].

Given that immune parameters are differentially regulated, measuring multiple humoral parameters can give a broad picture of the physiological immunocompetence of an organism [41]. Therefore, the immune response was evaluated in the present study by measuring phenoloxidase (PO) activity, nitric oxide (NO) production, antimicrobial activity, antimicrobial peptide transcripts (AMPs), and bacterial persistence. PO is an oxidoreductase used for cuticle melanization, wound repair, cytotoxin production and melanotic encapsulation [42, 43]. NO is a highly reactive and unstable free radical that inhibits the catalytic activity of enzymes and has damaging effects on the DNA of pathogens [44, 45]. Host AMPs produced soon after recognition of a foreign molecule have efficient antimicrobial activity [46]. Post-injected bacterial persistence was measured as a measure of the success of the immune response in male and female adults. The results are discussed within the perspective that sexual differences found in immune parameters revolve around natural selection for females and sexual selection for males, and that the degree of dimorphism depends on male and female life-history strategies.

## Materials and Methods

The colony of mosquitoes used in the present study was originally collected 10 years ago in the city of Cuernavaca, Morelos State, Mexico. With over 2000 individuals per generation, it has been propagated through random mating. Larvae and adult mosquitoes were herein reared under insectary conditions (12:12h light/dark cycle at 25–26°C) at the Instituto Nacional de Salud Pública (INSP). Fourth instar larvae and 3–5 day-old adult mosquitoes of both sexes were used for the experiments. For larval and adult challenges, we used live *Escherichia coli* (the 01268 strain, which is ampicillin resistant, was kindly donated by Dr. Jesús Silva, INSP-México), which was incubated in LB-broth at 37°C (200 RPM) for 3.25 h to reach the exponential growth phase (EGP).

### Bacterial exposure to larvae

**Fig 1:**
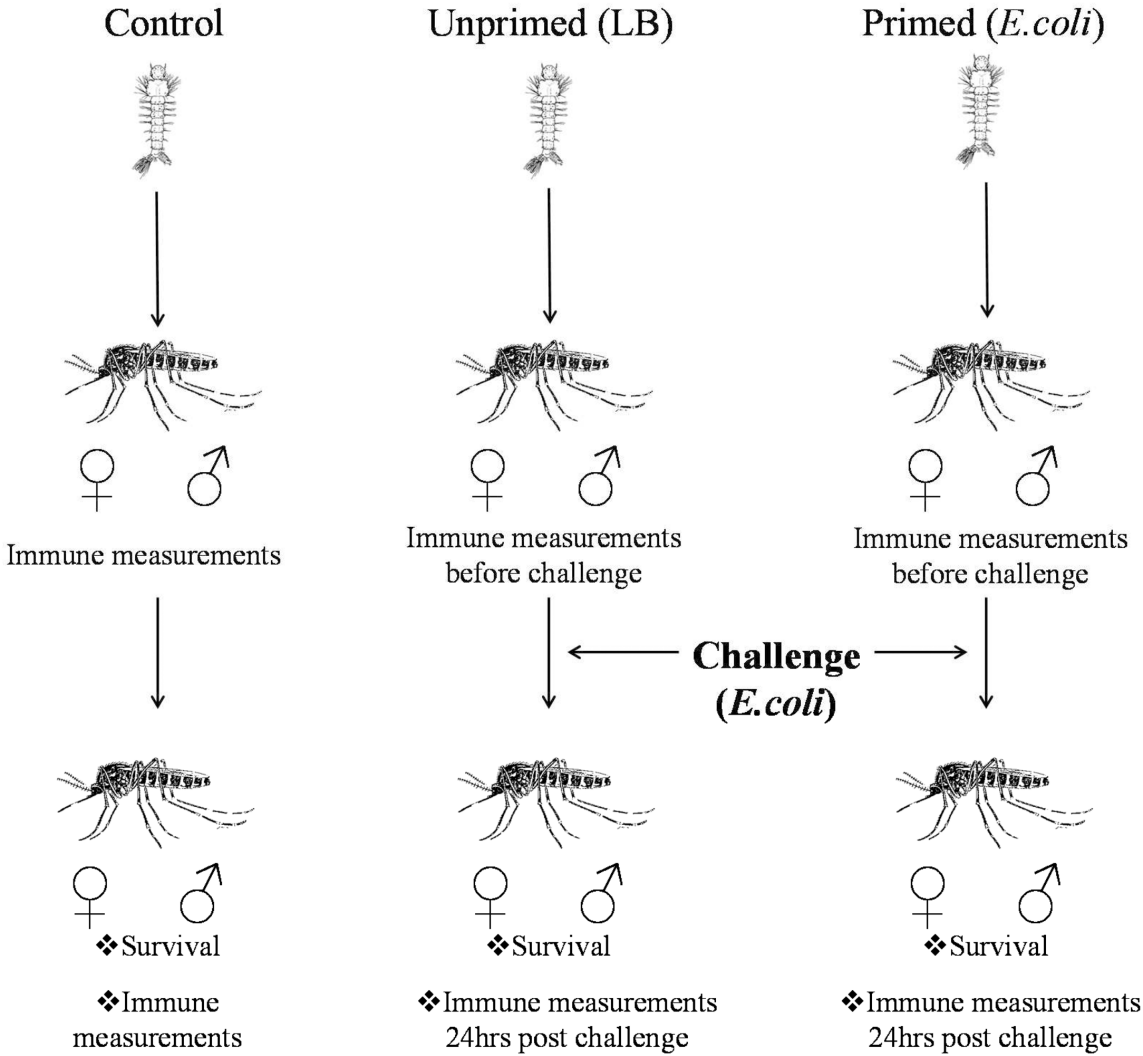
Experimental design. Induction of priming in fourth instar larva of *Aedes aegypti* against *E*. *coli*, and the measured parameters in adults. For both genders, parameters were recorded in adults both before the challenge and 24 h post-challenge. Survival was quantified after this challenge.

For the primed group (Pr), 4^th^ instar larvae were placed in LB broth mixed with *E*. *coli* (25 μl ≈ 1.7x10^6^ CFU of EGP bacteria + 175μl of LB; 200μl final volume per larva) for 1 h at room temperature. The use of LB instead of water was to prevent bacterial osmotic lysis. This bacterial concentration and time of exposure did not induce larva mortality. For the unprimed group (UnPr), 4^th^ instar larvae were placed in LB broth (200μl per larva) without bacteria, also for 1 h at room temperature. The control (C) group was maintained in water for 1 h (200μl per larva). For each group (Fig 1), 200 larvae were placed in 500 ml glasses. After the exposure period, the larvae of each group were washed three times with water before being placed in plastic containers with 2 liters of water, where they were left until the adults emerged. Then each group of adults was placed in a separate plastic container and allowed to feed *ad libitum* on cotton soaked in sugar solution.

### Adult mosquito challenge

Live EGP *E*. *coli* were injected in the abdomen (close to the junction between the ventral and dorsal cuticles) of adult mosquitoes of the UnPr and Pr groups. A pulled glass needle and a Drummond Captrol III microinjector were utilized to inject ~0.1μl with 7500 CFU of live *E*. *coli*. Mosquitoes were anaesthetized at 4°C for ten minutes previous to the injection and transferred to the insectary after the injection. Two independent assays were conducted for all experiments.

For all immune measurements, organisms from each group (C, UnPr, Pr) were collected 3–5 days after the adults emerged and 24 h post-challenge, and then stored at -70°C to await further processing. The pre-challenge sample gave information about the effect of the bacterial exposure at the larval stage on the immune condition of the emerging adult, while the post-challenge sample revealed the effect in the immune response that may have resulted from priming.

### Survival of adults after the challenge

Primed (N = 80 for females, N = 59 for males) and unprimed (N = 65 for females, N = 58 for males) adult mosquitoes were anaesthetized (at 4°C) and then injected with live *E*.*coli* (7500 CFU). RPMI (GIBCO-Na_2_CO_3_, 300mg/L L-glutamine) was used as a vehicle because mosquito mortality attributable to injection is reduced with this cell culture medium (Hernández-Martínez, pers. observ.). The control group (N = 45 for females, N = 73 for males) was only anaesthetized (for about 10 min). Mosquito mortality was recorded for 35 days.

### PO activity and NO production

Adult mosquitoes of each group (C, UnPr, and Pr, before and after adult challenge) were homogenized with a biovortexer in 130 μl of PBS buffer. Each sample was centrifuged for 10 min at 10,000 rpm (4°C). The supernatant was used to measure protein load concentration, PO activity and NO production. For PO and NO assays, a group of three mosquitoes was required to produce a sample because a single individual cannot provide enough sample for accurate spectrophotometer readings (OD >0.1).

Protein concentrations were normalized prior to the PO measurements. The BCA (Pierce) assay kit was used to determine protein concentration for each sample. The supernatant of normalized samples plus PBS were gauged at 50μl, mixed in a 96-microwell plate with 50μl L-DOPA (L-dihydroxyphenylalanine; 4mg/ml) as substrate, and incubated for 10 min at room temperature (24°C). Fifty μl of buffer mixed with 50μl of L-DOPA was used as blank. Absorbance was recorded every 5 minutes at OD_490_ on a plate reader during 30 minutes. This method was previously standardized and used by Moreno *et al*. [30]. PO activity was defined as the slope of activity over time.

The Griess reaction was employed to determine NO concentration [47]. Fifty μl of supernatant from each sample were mixed with 50μl of 1% sulfanilamide and 50 μl of 0.1% naphthyl ethylenediamine in a 96-microwell plate and incubated for 10 min at room temperature (24°C). NO was quantified using a NaNO_2_ (1–100 μM) standard reference curve for each assay. Absorbance was recorded every 5 minutes at OD_540_ on a plate reader. The highest reading obtained in an interval of 30 min was defined as NO production (expressed as μM).

### Antimicrobial peptide transcripts. Real-time quantitative PCR analyses

Total RNA was extracted from ten whole adult mosquitoes from each group (C, UnPr, and Pr, before and after adult challenge) using 500μl Trizol reagent (Invitrogen), measuring RNA concentration with Nanodrop. We used 500 ng/μl total RNA for cDNA synthesis using the RevertAid Premium Reverse Transcriptase (Thermo Scientific). The resultant cDNA was quantified and normalized, and 1μl was used for real-time quantitative PCR reactions. The qPCR reaction was performed using gene-specific primers for cecropin (*CEC* Id: AAEL015515-RA, 160 pb; forward 5´ TCA CAA AGT TAT TTC TCC TGA TCG 3´; reverse 5´ GCT TTA GCC CCA GCT ACA AC 3´), attacin (*ATA* Id: AAEL003389-RA, 231 pb; forward 5´ TTG GCA GGC ACG GAA TGT CTT G 3´; reverse 5´ TGT TGT CGG GAC CGG GAA GTG 3´), defensin (*DEF* Id: AAEL003832-RA, 200 pb; forward 5´ TTG TTT GCT TCG TTG CTC TTT 3´; reverse 5´ ATC TCC TAC ACC GAA CCC ACT 3´) and *ribosomal protein S7* (internal control, Id: AAEL009496-RA, 190 pb; forward 5´ GGG ACA AAT CGG CCA GGC TAT C 3´, reverse 5´ TCG TGG ACG CTT CTG CTT GTT G 3´), and Maxima SYBR Green/ROX qPCR Master Mix (Thermo Scientific) on a StepOne Plus Real-Time PCR System (Applied Biosystem). These sequences have been previously used in *Ae*. *aegypti* [48].

Relative quantification of mRNA levels was done by the 2^-ΔΔC^_T_ method, and primer efficiencies were calculated by measuring how the standard ΔC_T_ varies with five template serial dilutions. For all trials, the ribosomal protein gene S7 was used as a reference. The levels of CEC, DEF and ATA transcripts were normalized with respect to that of the S7 transcript found with the same sample. Melting curve analyses confirmed that only cDNA, and not genomic DNA, was amplified. The transcriptional induction of peptides in the unprimed and primed groups were evaluated relative to naïve controls both before injection of adults and 24 h post-injection. Three independent trials were conducted, each analyzed in duplicate.

### Antibacterial activity

Individual mosquitoes of each group (C, UnPr, and Pr, before and after adult challenge) were pestled with a biovortexer in 50 μl of sterile PBS buffer, and each sample was centrifuged for 10 min at 10,000 rpm (4°C). In 96-microwell plates, 50 μl of the same *E*. *coli* (EGP) used for exposure (at a starting OD_620_ of 0.001nm) was cultured in 50 μl of each sample and incubated at 30°C. Absorbance readings were recorded every hour at OD_620_ during 6 h, with a final reading at 24 h.

### Bacterial persistence in adult mosquitoes

Bacterial persistence was measured to determine the intensity of bacterial elimination after exposure at the larval stage and 24 h after injection of the adults. Live male and female mosquitoes from each group (C, UnPr, and Pr, before and after adult challenge) were cold anesthetized at 4°C for 10 min (for this assay, mosquitoes were not placed at -70°C to avoid the death of the remaining bacteria), then placed in 70% alcohol for 30 seconds to eliminate bacteria present on the cuticle. After alcohol evaporation, each mosquito was placed in a 0.6 ml microtube. Mosquitoes were homogenized with a biovortexer in 50 μl of sterile PBS buffer. Twenty μl of the macerate were then placed in culture tubes with 4ml of LB/ampicillin (150μg/ml). After 6.5 h of incubation at 37°C (shaken at 200 rpm), period necessary for the *E*. *coli* used under these conditions to reach accurate absorbance readings, 200 μl from each tube were placed in each well of a 96-microwell plate, and turbidity was measured at OD_620_. The turbidity intensity of the culture expressed the population of bacteria.

### Data analysis

A *Log-rank x* ^*2*^ test was used to detect differences in survival curves among the C, UnPr and Pr groups for both male and females. Survival analyses were undertaken using JMP 7.0 (SAS Institute, 2004). For all immune measurements, the differences between treatments (C, UnPr, and Pr, before and after adult challenge) and males/females were analyzed with two-way analysis of variance (ANOVA) by using the *F*-Test. Where significant ANOVA differences were found, an LSD Fisher Post-Hoc test was employed to identify the nature of these differences (*P<*0.05 for significance). For bacterial persistence, a repeated measure two-way ANOVA (GLM module of Statistica) was used to analyze the differences between treatments (C, UnPr, and Pr, before and after adult challenge) and males/females, followed by an LSD Post-Hoc analysis when appropriate. Data from the immune measurements (PO, NO, antimicrobial activity, and qPCR) were tested for normality (Shapiro-Wilks Normality test) and homogeneity variance prior to any further analysis. Only for antimicrobial activity was data Ln transformed to achieve normality. All values are presented as the mean ± SE. Statistical analyses were undertaken using Statistica (StatSoft, Inc). Details can be found in S1 Dataset.

## Results

### Survival

**Fig 2:**
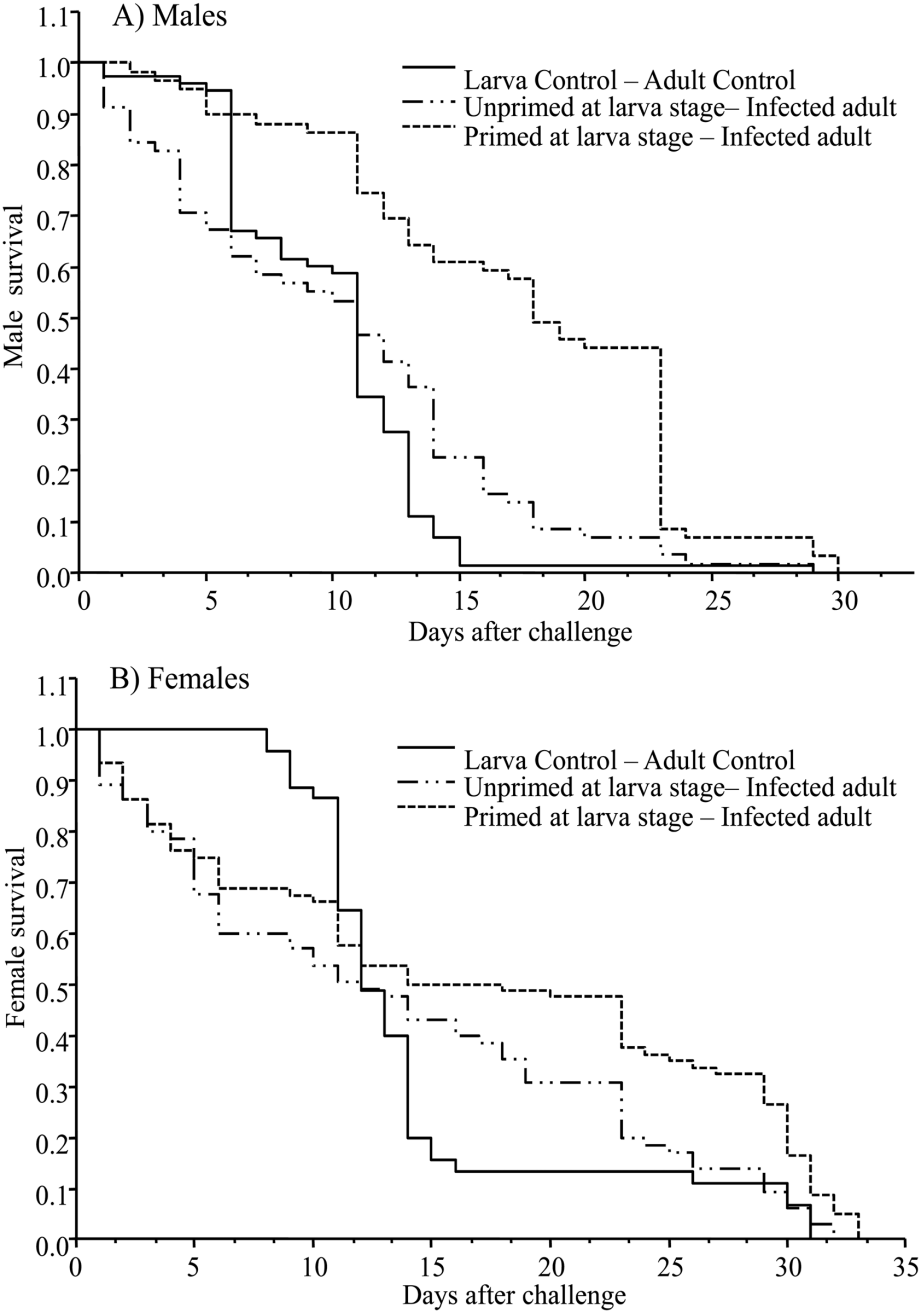
Survival curves of mosquitoes of the C, UnPr, and Pr groups after the challenge in adults. (A) Males and (B) females.

The survival analyses revealed that compared to unprimed mosquitoes, exposure of fourth larva instar with *E*. *coli* provided protection to adult males (*Log-Rank x* ^*2*^ = 48.018, *df* = 2, *P* < 0.0001) and females (*Log-Rank x* ^*2*^ = 7.9046, *df* = 2, *P* = 0.0192) when they were re-exposure (injected) with the same bacteria. Removal of the C group from the survival model did not change the significant differences (Fig 2) (see also S1 A-G Fig). The protective effect of priming was greater in males than females (Fig 2A and 2B).

### PO activity

**Fig 3:**
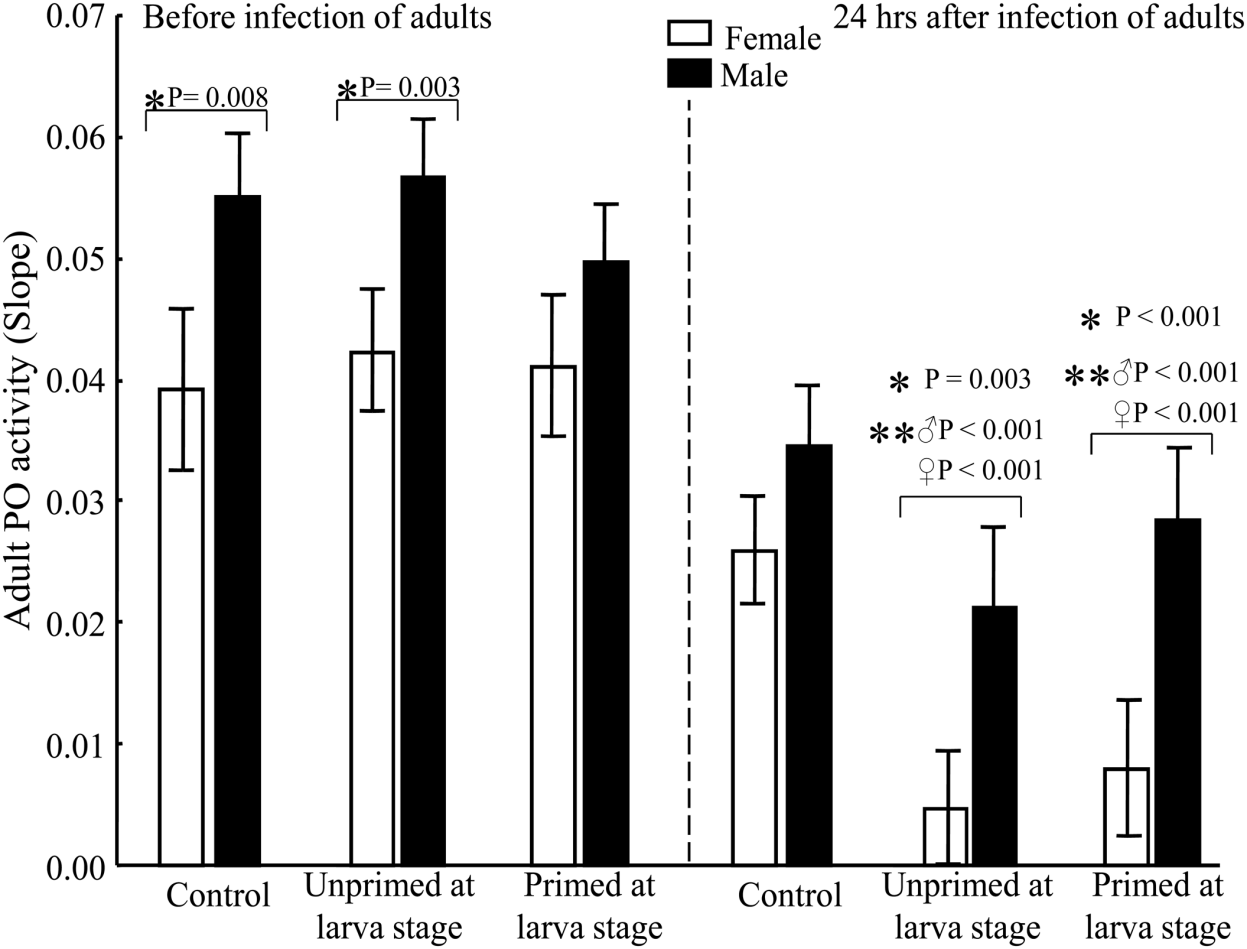
Gender differences for the PO activity. (*) indicates a significant difference between genders within a treatment group (C, UnPr, Pr). (**) indicates a significant difference between the measurements before and after infection of adults (for the C group at 24 h post-challenge, the significant difference was only found for males). Number of samples per group before infection: ♀ = 14, ♂ = 24 for C; ♀ = 24, ♂ = 26 for UnPr; ♀ = 18, ♂ = 26 for Pr. Number of samples per group 24 h after infection: ♀ = 30, ♂ = 26 for C; ♀ = 28, ♂ = 14 for UnPr; ♀ = 19, ♂ = 18 for Pr.

No differences were observed in the value of PO between the UnPr and Pr groups, either before or after the adult challenge (Table 1). Nevertheless, we found an overall difference between males and females, with the former showing a higher PO activity (*F*_(1, 5)_ = 78.712, *P* < 0.0001). This difference was statistically significant in almost all groups (LSD Post-Hoc, *P* < 0.005; Fig 3; Table 2). Interestingly, within each group there was a lower PO activity 24 hours after the challenge than before the challenge (LSD Post-Hoc, *P* < 0.0001), which was more pronounced in females. Moreover, compared to the uninjected control group, there was lower PO activity after injection with bacteria.

**Table 1:** Immune response of adult males and females when previously primed at the larval stage, compared to the groups not exposed to *E*.*coli* (unprimed) at the larval stage. A) Response of emerged adult; B) response of adults after infection with *E*. *coli*.

	A) Immune priming (Unprimed vs. Primed group) in emerged adults		B) Immune priming (Unprimed vs. Primed group) at 24 h post-infection of adults	
	*Females*	*Males*	*Females*	*Males*
**PO activity**	N.D.	N.D.	N.D.	N.D.
**NO production**	N.D.	N.D.	N.D.	N.D.
**AMPs transcripts**	N.D.	N.D.	Higher in the primed group: Attacin and ceropin, *P*<0.001; Defensin, *P* = 0.044	N.D.
**Antimicrobial activity**	Higher in the primed group, *P*<0.001	N.D.	Higher in the primed group, *P* = 0.003	N.D.
**Bacterial persistence**	N.D.	N.D.	Higher in the primed group, *P*<0.001	N.D.

N.D. = no differences between groups.

**Table 2:** Gender-based immune differences among: a) emerged adults, and b) adults at 24 h post-infection.

	A) Emerged adult			b) Adult at 24 h post-infection		
	*Control group*	*Unprimed at larval stage*	*Primed at larval stage*	*Control group*	*Unprimed at larval stage*	*Primed at larval stage*
**PO activity**	Higher in males, *P* = 0.008	Higher in males, *P* = 0.003	-	-	Higher in males, *P* = 0.003	Higher in males, *P*<0.001
**NO production**	-	Higher in females, *P* = 0.004	Higher in females, *P* = 0.002	-	-	-
**AMP transcripts**	-	-	-	-	Defensin higher in females, *P* = 0.03	Defensin higher in females, *P*<0.001
**Antimicrobial activity**	Higher in females, *P*<0.001	Higher in females, *P*<0.001	Higher in females, *P*<0.001	Higher in females, *P*<0.001	Higher in females, *P* = 0.003	Higher in females, *P*<0.001
**Bacterial persistence**	-	-	-	-	-	Higher in females, *P* = 0.003

### NO production

**Fig 4:**
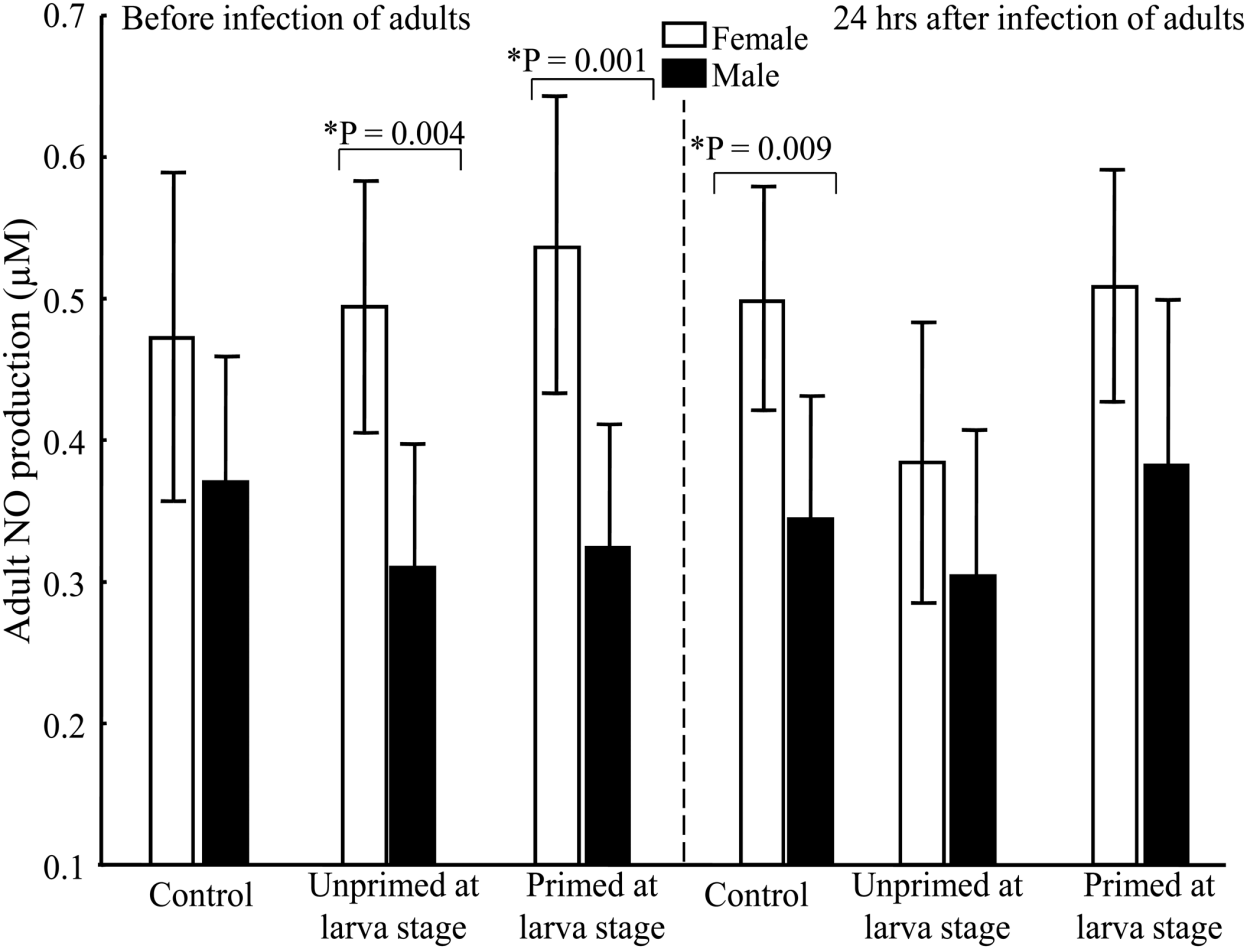
Gender differences in nitric oxide production (expressed as μM). (*) indicates a significant difference between genders within treatment groups. Three mosquitoes were used to obtain a single sample. Number of samples per group before infection: ♀ = 14, ♂ = 24 for C; ♀ = 24, ♂ = 26 for UnPr; ♀ = 18, ♂ = 26 for Pr. Number of samples per group 24 h after infection: ♀ = 30, ♂ = 26 for C; ♀ = 28, ♂ = 14 for UnPr; ♀ = 19, ♂ = 18 for Pr.

No differences were observed in the value of NO between the UnPr and Pr groups, either before or after the adult challenge (Table 1). However, statistically significant differences between adult males and females were detected within the UnPr and Pr groups before the challenge, and between the Pr and C groups at 24 h post-challenge (*P* > 0.01; Fig 4; Table 2). Additionally, we observed a tendency to a higher NO production in the Pr than UnPr group at 24 h post-challenge, but this difference was not statistically significant.

### Antimicrobial peptide transcripts

**Fig 5:**
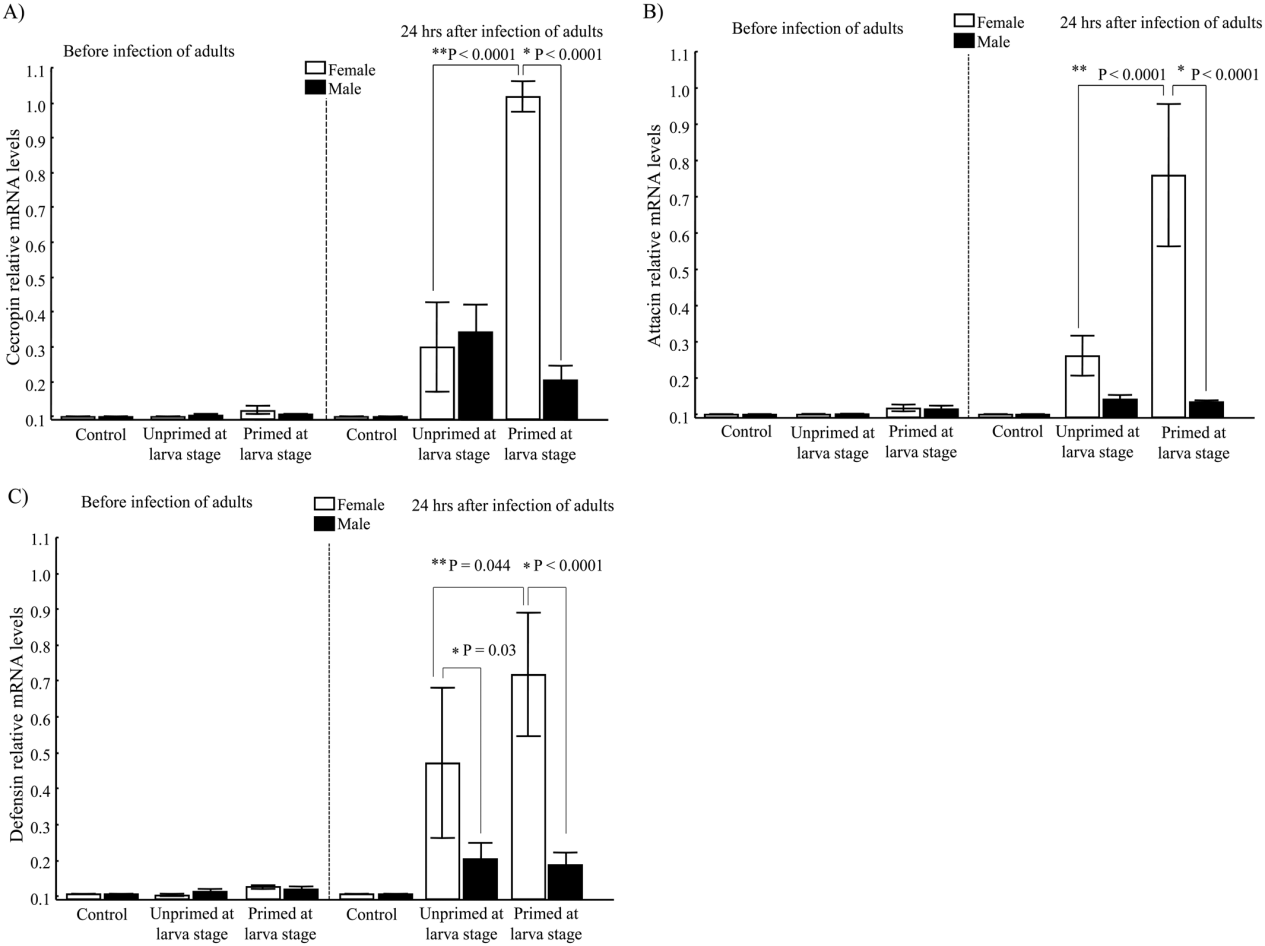
Peptide transcription in adults of both sexes, with or without priming induced at the larval stage. (A) Analysis of transcriptional levels through quantitative RT-PCR before and after infection of adults, measuring cecropin, (B) Analysis of transcriptional levels through quantitative RT-PCR before and after infection of adults, measuring attacin, (C) Analysis of transcriptional levels through quantitative RT-PCR before and after infection of adults, measuring defensin. (*) indicates a significant difference between males and females, and (**) indicates a significant difference between the UnPr and Pr groups.

Before the challenge, no differences in relative mRNA levels of the transcripts were observed for either of the three peptides (Fig 5A, 5B and 5C). However, 24 h post-challenge, cecropin transcripts were higher in both the UnPr and Pr groups compared to the C group, and in the females versus males of the Pr group. The latter result indicated sexual dimorphism in the primed group (LSD Post-Hoc, *P*<0.0001; Fig 5A; Table 1).

We also observed a priming effect in females with regard to attacin and defensin, evidenced by the higher level of these peptides found for this gender in the Pr than UnPr group (LSD Post-Hoc, *P*<0.0001 and *P* = 0.044, respectively; Fig 5B and 5C). Whereas sexual dimorphism was observed in relation to attacin only between males and females of the Pr group (LSD Post hoc, *P*<0.0001; Fig 5B; Table 2), significant gender differences were found for defensin in both the UnPr and Pr groups (LSD Post-Hoc, *P* = 0.03 and P<0.0001, respectively; Fig 5C). In all of these cases, higher transcript expression was found in females.

### Antibacterial activity

**Fig 6:**
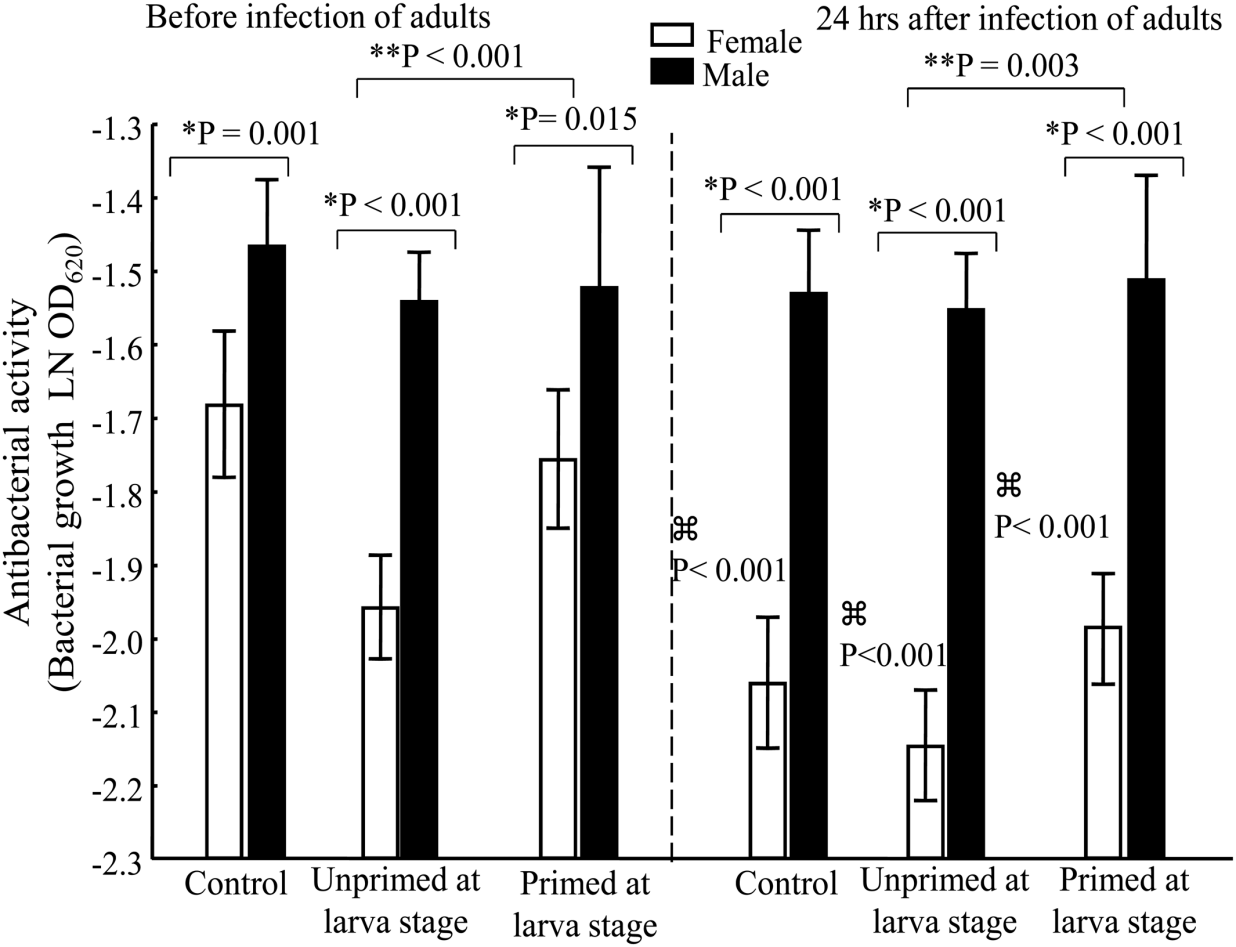
Antibacterial activity of adult mosquitoes before and after infection with *E*. *coli*. Higher readings indicate weak antibacterial activity. (*) indicates a significant difference between genders within each treatment group (UnPr, Pr). (**) indicates a significant difference between the UnPr and Pr groups (only for females). (⌘) indicates a significant difference between measurements before and after infection of adults (only for females). Number of samples per group before infection: ♀ = 7, ♂ = 10 for C; ♀ = 16, ♂ = 18 for UnPr; ♀ = 11, ♂ = 3 for Pr. Number of samples per group after infection: ♀ = 10, ♂ = 11 for C; ♀ = 14, ♂ = 14 for UnPr; ♀ = 14, ♂ = 4 for Pr.

Antibacterial activity was significantly greater in females than males for all groups (overall ANOVA *F*_(1, 119)_ = 207.62, *P* < 0.0001; Fig 6; Table 1). Before and after the adult challenge, there was a significant effect on females produced by the bacterial exposure at the larval stage (LSD Post-Hoc, *P* < 0.005; Table 2). There was a tendency to greater antibacterial activity in the Pr versus UnPr group, but it was not statistically different (Fig 6).

### Bacterial persistence

**Fig 7:**
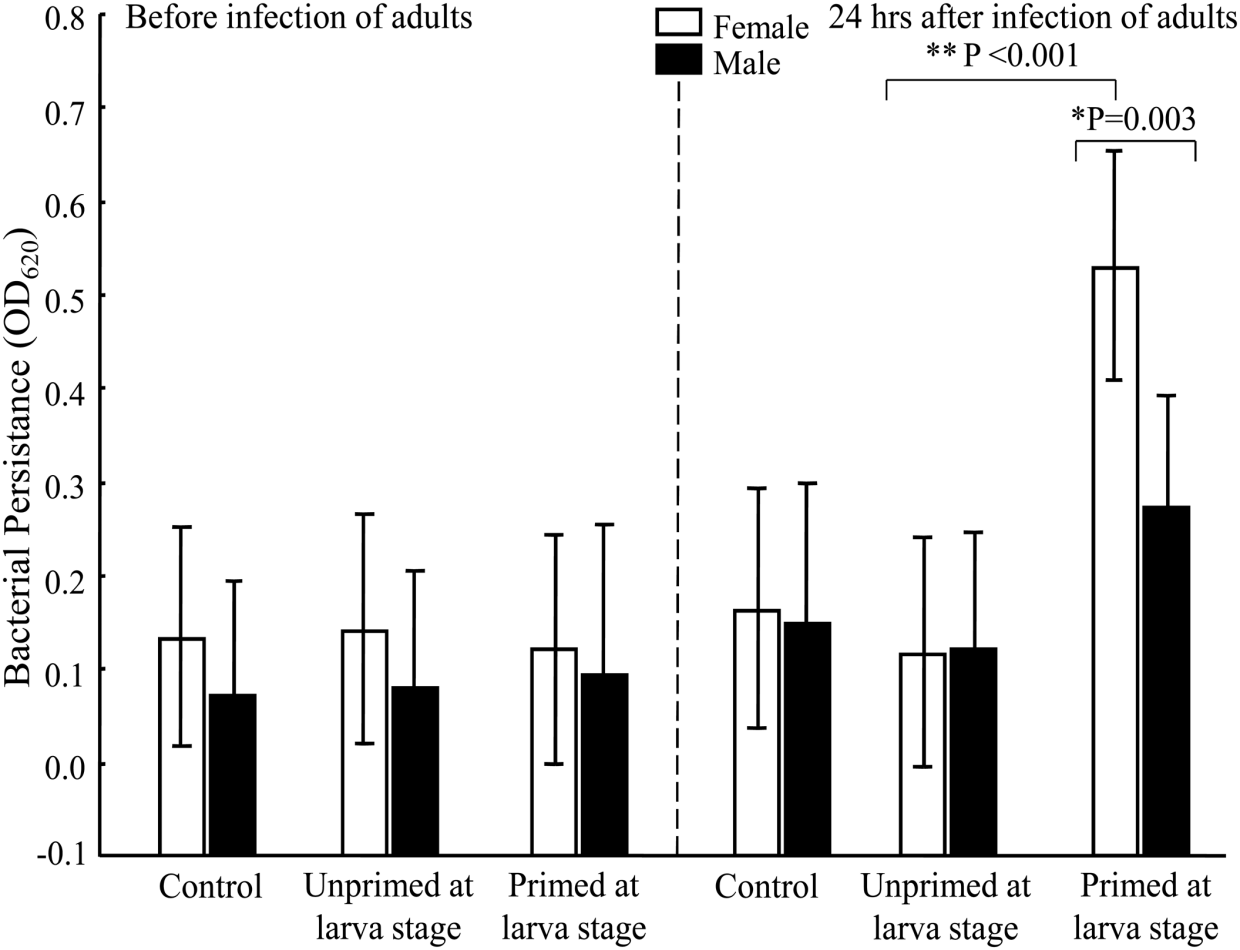
*E*. *coli* persistence after infections. Persistence was defined as the turbidity intensity of culture in LB. (*) indicates a significant difference between genders within each treatment group (C, UnPr, Pr); (**) indicates a significant difference between UnPr and Pr females 24 h after infection of adults. Number of samples per group before infection: ♀ = 10, ♂ = 6 for C; ♀ = 11, ♂ = 10 for UnPr; ♀ = 10, ♂ = 10 for Pr. Number of samples per group after infection: ♀ = 9, ♂ = 7 for C; ♀ = 10, ♂ = 10 for UnPr; ♀ = 10, ♂ = 10 for Pr.

Bacterial load quantification revealed that *E*. *coli* did not persist from larva that were infected to adult male or female mosquitoes (considering the Pr group before the challenge; Fig 7). However, after the challenge in adults, bacterial persistence was greater in females of the Pr than UnPr group (LSD Post-Hoc, *P*< 0.0001; Table 1). Moreover, in the Pr group there was less bacterial persistence in males than females after the adult challenge (LSD Post-Hoc, *P*< 0.003; Table 2).

## Discussion

Contrary to the common idea that the immunity of females is superior to that of males, the results of the current contribution show that when *Ae*. *aegypti* mosquitoes are primed at the larval stage, the survival of both adult males and females is enhanced (compared to the unprimed group) after a bacterial challenge. This increased survival time for males was found in spite of the shorter life span in this mosquito specie for males than females [49]. This enhancement could be explained only if male survival is as important as that of females.

Because of their role in reproduction, it is assumed that males reduce their investment in the immune defense to increase reproductive effort [25], and that females increase their investment in immunity to achieve improved longevity and therefore greater reproductive success [27]. However, the findings of Ponlawat and Harrington [50] suggest an advantage in *Ae*. *aegypti* for the longevity of males as well, evidenced by the greater production and storage of sperm in the reproductive organs of older (over 10 days old) than younger (under 10 days old) males (this production was associated with increased sperm transference to females).

Hence, in this species priming apparently offers a reproductive pay-off by increasing survival and therefore leading to greater reproductive opportunities through an increased gamete transference. It is then possible that older males of this species expend greater resources on reproduction, but this is only feasible if they are at a low risk for bacterial (or any other) infection. For females, increased longevity could maximize the length of the reproductive period, as proposed by Bateman’s principle, a possibility that needs to be tested in this species.

Although the mechanisms of immune priming in invertebrates remain unclear, receptor molecules such as Dscam [51], C-lectins [52] and epigenetic mechanisms could promote innate immune memory in cells or tissues for a more enhanced secondary response in invertebrates [53]. The protection observed in both males and females of the present study could have been a consequence of increased pathogen recognition acquired during priming, which then boosted activation of immune signaling pathways when a second infection occurred (see [15]). Since the injected groups showed greater survival than the control group, the immune molecules induced by injection could have had a protective action against other possible infections. Further research is needed on this question.

Although none of the immune response factors measured herein explain the enhanced survival of primed males, they do indeed reveal distinctive gender protective strategies used against bacterial infection. Whereas females showed higher NO production than males, males exhibited greater PO activity than females. In fact, there was a significant reduction in PO activity in both genders, being greater in females. However, neither if these molecules was correlated with a priming effect.

The present findings are similar to previous reports comparing males and females of this mosquito specie in which males had greater PO activity and females had two-fold higher production of NO [30]. The current results are also consistent with the idea that traits involved in the immune response do not have the same importance for males and females [54]. Lower PO activity in females could be explained if resources are allocated to reproductive activity (egg production) rather to the maintenance of the PO cascade. The greater PO activity in males may be associated with their environmental conditions or some sexual traits, as is the case with some other insects [55]. On the other hand, the higher NO production in females is likely related to the essential nature of this molecule for the protective response against bacteria in the hemocoel of this gender [56]. Additionally, NO contributes in the response against DENV [57].

The proximate mechanisms that shape SID are not yet understood. Gender differences in immunity could arise from dissimilarities in physiology (metabolic rate, food, efficiency of conversion), expression of juvenile hormone, and behavior (food consumption). An often neglected aspect of the hypothesis of sexual differences in immunity is the difference in the microbial load or in the types of pathogens attacking each sex. Unlike males, for example, female mosquitoes are exposed to a large amount of bacteria in their gut during blood digestion. On the other hand, a distinct immune response should be expected for males and females given that each gender confronts distinct pathogens and trade-offs.

The antibacterial activity analysis of the present study also showed dimorphism in the immune response, in this case in the expression of functional molecules that limit pathogen growth. Females had a higher antibacterial activity than males before and after the adult challenge. However, despite the higher antibacterial activity found in Pr females than Pr males, the former were incapable of clearing bacteria. Furthermore, previous infection at the larval stage did not cause an enhanced antibacterial activity in female adults. The AMP transcripts reveal the contrasting effects on males and females of priming induced at the larval stage. A priming effect was only observed in relation to Pr females after the adult challenge, evidenced as an increase in the levels of antimicrobial peptide transcripts.

As mentioned previously, males and females use a similar genome to produce phenotypes. For example, only a small fraction of the transcriptome of *Drosophila melanogaster* displays sex-dependent regulation [58]. However, a gene can have variants that confer a slight advantage in expression for males or females, a fact that can explain the differential transcription observed in the present study between the three AMPs analyzed and between genders. Selective pressures could induce antagonistic evolutionary forces for males and females, giving rise to an intersexual conflict [59]. This conflict may also occur with immune traits that require prolonged and coordinated development in order to reach the functional phenotype at the adult stage. However, this idea requires further research.

The results of antibacterial activity, bacterial persistence and AMP transcripts seem to contradict one another if we assume that AMPs have a cytotoxic effect. However, AMPs sometimes protect the insect against the bacteria that persist rather than clearing bacterial infections [60]. Bartholomay et al. [61] proposed that defensins may be elements of the stress response or have a chemotactic function. Meanwhile, cecropin and attacin have insufficient activity against Gram-negative bacteria [62–65]. Therefore, the increased transcriptional level of some peptides observed herein does not necessarily correlate with a greater killing activity, but may instead be related to a protective state. The idea of a protective effect is in concordance with the results of bacterial persistence in this study—bacteria was removed by males but not females of the UnPr and Pr groups. Whereas pathogen clearance/resistance may be an adaptive defense strategy for males, tolerance (which is energetically low-cost, but efficient against pathogens) could be the preferred strategy for females.

We are aware that mosquitoes employ both a molecular and cellular response to bacteria [66], and that the present study focuses only on the humoral response. Hemocytes are the first line of defense for controlling bacteria [67–69] and are correlated with priming [15, 16]. Therefore, we cannot exclude the possibility that the enhanced survival of males also depended on cellular activity and/or other humoral molecules not measured herein. This is another area that requires further research. The present results led us to consider that not all the immune effectors undergo a simultaneous increase in response to pathogens. Rather, it seems likely that there is a coordinated response to pathogens that can limit the bacterial infection and thereby enhance mosquito survival.

Research on mosquitoes usually analyzes the adult stage. Nevertheless, conditions experienced at immature stages are equally important because this early ontogenetic period influences the adult immune response to infection. As mentioned before, it is possible that epigenetic mechanisms could also be promoting innate immune memory in larva cells or tissues to induce an enhanced secondary response at adult stage. A recent study suggested that enhanced induction of endoreplication after the second exposure could be responsible for the priming effect in mosquitoes [70]. This mechanism could be also implicated in the observed priming in this study. Analysis of larva immune response against *E*.*coli* must be done to characterize the molecular mechanisms that allow immune priming to persist from immature to adult stage. This will let us know if immune responses at larval correlates with adult immunity. Previous studies on immune priming with *Ae*. *aegypti* [71, 72] have provided the paradigm of immunity based on the anticipated response. The current contribution (and a previous ones [13, 21, 22]) adds another dimension to immune priming by providing a primary characterization of the gender-based and complex immune response of mosquitoes against a bacterial challenge after previous priming at an earlier ontogenetic stage.

Finally, SID evidenced by the present results suggests a possible gender-based strategy of *Plasmodium* in its mosquito vector, because survival of the female host is essential for pathogen transmission. How long a female mosquito can survive determines the number of times it can reproduce and whether or not it is available to repeatedly feed on blood in order to transmit the pathogen from one host to another [73]. Immune priming may be beneficial for the mosquito, thus increasing the vectorial capacity with an adverse impact on public health.

## Supporting Information

S1 Fig Pairwise survival curves of mosquitoes of the C, UnPr, and Pr groups after the challenge in adults. **S1A Fig** Survival differences between infected groups of males that were previously exposed to bacteria (primed males) and not exposed (unprimed males) to bacteria at larval stage. The Control group was removed. **S1B Fig** Survival differences between infected groups of females that were previously exposed to bacteria (primed females) and not exposed (unprimed females) to bacteria at larval stage. The Control group was removed. **S1C Fig** Survival differences between males and females that were primed at larval stage and then infected at adult. The Control groups were removed. **S1D Fig** Survival differences between Control and Primed males and females (that were injected with bacteria at adult stage). **S1E Fig** Survival differences between Control and Primed females (that were injected with bacteria at adult stage). **S1F Fig** Survival differences between infected males and females that were not exposed to bacteria (unprimed groups) at larval stage. The Control group was removed. **S1G Fig** Survival differences between adult males and females (control groups).
(DOCX)

S1 Dataset Immune responses dataset. (XLSX)
