# Supplementary material for: Bacterial Exposure at the Larval Stage Induced Sexual Immune Dimorphism and Priming in Adult Aedes aegypti Mosquitoes
Source: PLoS One. 2015 Jul 16;10(7):e0133240. doi: 10.1371/journal.pone.0133240 (PMC4504673; doi:10.1371/journal.pone.0133240)
Supplement: S1 Fig — S1A Fig Survival differences between infected groups of males that were previously exposed to bacteria (primed males) and not exposed (unprimed males) to bacteria at larval stage. The Control group was removed. S1B Fig Survival differences between infected groups of females that were previously exposed to bacteria (primed females) and not exposed (unprimed females) to bacteria at larval stage. The Control group was removed. S1C Fig Survival differences between males and females that were primed at larval stage and then infected at adult. The Control groups were removed. S1D Fig Survival differences between Control and Primed males and females (that were injected with bacteria at adult stage). S1E Fig Survival differences between Control and Primed females (that were injected with bacteria at adult stage). S1F Fig Survival differences between infected males and females that were not exposed to bacteria (unprimed groups) at larval stage. The Control group was removed. S1G Fig Survival differences between adult males and females (control groups). (DOCX) [file pone.0133240.s001.docx]

Supplementary Figure 1.

**Fig. A)**

**Fig. B)**

**Fig. C)**

**Fig. D)**

**Fig. E)**

**Fig. F)**

**Fig. G)**
